# Impact of Reversion of *Mycobacterium tuberculosis* Immunoreactivity Tests on the Estimated Annual Risk of Tuberculosis Infection

**DOI:** 10.1093/aje/kwad028

**Published:** 2023-02-07

**Authors:** Alvaro Schwalb, Jon C Emery, Katie D Dale, Katherine C Horton, César A Ugarte-Gil, Rein M G J Houben

**Keywords:** interferon-γ release assay, *Mycobacterium tuberculosis* transmission, TST/IGRA surveys, tuberculin skin test, tuberculosis

## Abstract

A key metric in tuberculosis epidemiology is the annual risk of infection (ARI), which is usually derived from tuberculin skin test (TST) and interferon-γ release assay (IGRA) prevalence surveys carried out in children. Derivation of the ARI assumes that immunoreactivity is persistent over time; however, reversion of immunoreactivity has long been documented. We used a deterministic, compartmental model of *Mycobacterium tuberculosis* (Mtb) infection to explore the impact of reversion on ARI estimation using age-specific reversion probabilities for the TST and IGRA. Using empirical data on TST reversion (22.2%/year for persons aged ≤19 years), the true ARI was 2–5 times higher than that estimated from immunoreactivity studies in children aged 8–12 years. Applying empirical reversion probabilities for the IGRA (9.9%/year for youths aged 12–18 years) showed a 1.5- to 2-fold underestimation. ARIs are increasingly underestimated in older populations, due to the cumulative impact of reversion on population reactivity over time. Declines in annual risk did not largely affect the results. Ignoring reversion leads to a stark underestimation of the true ARI in populations and our interpretation of Mtb transmission intensity. In future surveys, researchers should adjust for the reversion probability and its cumulative effect with increasing age to obtain a more accurate reflection of the burden and dynamics of Mtb infection.

This article is linked to 'Invited Commentary: The Winding Road to Identifying the Annual Rate of Tuberculosis Infection' and 'Schwalb and Houben Respond to ``The Winding Road to ARTI"' (https://doi.org/10.1093/aje/kwad125 and https://doi.org/10.1093/aje/kwad163).

## Abbreviations

ARI: annual risk of infection
CI: confidence interval
IGRA: interferon-γ release assay
Mtb: *Mycobacterium* *tuberculosis*
TB: tuberculosis
TST: tuberculin skin test

***Editor’s note:** An invited commentary on this article appears on page 1944, and the authors' response appears on page 1947.*

Tuberculosis (TB) remains a major cause of morbidity and mortality worldwide, and it is estimated that one-quarter of the global population is latently infected with *Mycobacterium tuberculosis* (Mtb) (1–3). Mtb infection is inferred from the presence of a host immune response to Mtb protein components with the use of the tuberculin skin test (TST) or interferon-γ release assay (IGRA) (4, 5). While it is known that Mtb immunoreactivity does not equate to Mtb infection, population surveys of TST positivity have historically been used to derive estimates of Mtb infection risk and transmission trends, most conducted among school-age children (ages 8–12 years) (6). A key metric in TB epidemiology is the annual risk of infection (ARI), which aims to provide a more insightful picture of the risk of Mtb transmission (7). It is calculated using Mtb immunoreactivity test prevalence data and the mean age of the individuals surveyed (8). In a public health setting, a decrease in the ARI is interpreted as an early indicator of a decline in Mtb transmission in a population; on the other hand, an increase could indicate that TB prevention and care measures are insufficient (8).

When calculating ARIs, there is a conventional, usually implicit, assumption that positive Mtb immunoreactivity is persistent throughout an individual*’*s lifetime (9). Nevertheless, this assumption does not hold. TB immunoreactivity can wane over time, and positive TSTs and IGRAs can revert to negative (reversion) (10–12). Therefore, a major caveat in the ARI is that the phenomenon of reversion is not accounted for in its calculation, thus resulting in a naive ARI which might differ from the true value. In previous studies, investigators have considered the limitations of the current formula in arriving at an accurate estimate and interpretation of the ARI (10, 13). In a theoretical study by Sutherland (13), the effects of TST reversion on the ARI were explored, suggesting a considerable underestimation when annual reversion probabilities exceed 1% (nearly 50% and 67% when facing annual reversion probabilities of 5% and 10%, respectively). However, the Sutherland study considered only low reversion probabilities (≤10%); it did not consider age-specific effects, nor did it link to observed reversion data. While empirically observed reversion probabilities were documented over a century ago (14), their importance has been largely dismissed. With a few notable exceptions, immunoreactivity surveys do not usually consider reversion when estimating the ARI (10, 12). This is an issue, because ARI estimates without consideration of reversion are likely to underestimate the proportion of individuals once infected with Mtb (8).

In contemporary policy, the ARI remains important and is estimated in TST/IGRA surveys in populations or high-risk settings (15–17). The ARI is also a common parameter in the mathematical modeling of TB—for example, to estimate the global burden of latent Mtb infection or to set the intensity of transmission in a population (3, 18). Moreover, as novel diagnostic tools that measure Mtb immunoreactivity become available and immunoreactivity surveys may be reconsidered in global policy, it is important to consider the reversion level of specific tests so ARI underestimation can be appropriately quantified through current methods. In this paper, we aimed to use empirical estimates of reversion for TST and IGRA to quantify the extent of ARI underestimation due to reversion.

## METHODS

### Model overview

**Figure 1:**
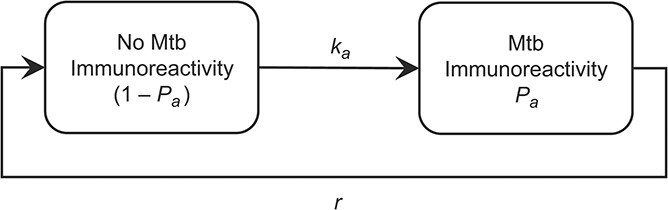
Model of *Mycobacterium tuberculosis* (Mtb) immunoreactivity accounting for reversion. *k_a_* represents real infection risk, which is a function of the annual risk of infection (ARI) at birth (ARI_0_), with a subsequent annual decrease in risk; *P_a_* represents the proportion of the population found to be Mtb-immunoreactive at age *a* years; and *r* represents the annual constant proportion of individuals with positive immunoreactivity who will revert.

We developed a deterministic, compartmental model of Mtb infection (Figure 1). It builds on the theoretical study on the effect of constant TST reversion probabilities upon the ARI estimation proposed by Sutherland (13). The proportion of the population found to be immunoreactive at age *a* years is expressed by *P_a_*. The parameter *k_a_* represents the real infection risk, a function of the ARI at birth (ARI_0_), with subsequent annual decrease *d* in risk: ${k}_a={\left(1-d\right)}^a\times{\mathrm{ARI}}_0$. Additionally, the model includes an annual constant proportion *r* of individuals with positive immunoreactivity who will revert. In order to estimate the proportion infected in the next year, the following formula is used:

$$ {P}_{\left(a+1\right)}={P}_a+\left(1-{P}_a\right)\times{k}_a-{P}_a\times r. $$

The formula has 3 components: 1) the proportion of the population infected with Mtb in the current year, 2) plus the proportion of noninfected individuals who convert to positive immunoreactivity over the following year, 3) minus the proportion of immunoreactive individuals who revert over the following year. For a fixed initial ARI of 1.5%, Mtb immunoreactivity prevalence was calculated in daily time steps using increasing reversion probabilities from 0% to 50% with 1% increments from birth to age 80 years. For all ages and Mtb immunoreactivity prevalence, the ARI was calculated using the classic formula ${\mathrm{ARI}}_a=1-{(1-{P}_a)}^{{^1}/_a}$. Then, the base ARI (not accounting for reversion) was compared against each reversion ARI (up to 50% reversion) as a ratio. Since we are considering that reversion is occurring but not accounted for in the calculation of the ARI, this ratio reflects how much the naive ARI must increase to match the measured prevalence, resulting in the true ARI. The model was constructed and the analysis run using R, version 4.1.0 (May 18, 2021) for statistical computing and graphics (19). Plots were created using the *ggplot2* package (20).

### Model assumptions

The key assumption of our model is that Mtb infection always leads to Mtb immunoreactivity, regardless of different cutoff values and incremental changes considered in conversion criteria (21). Furthermore, the model does not account for reinfection, assuming that reinfections occur at a similar rate as primary infections; this was done for simplicity. Finally, it assumes that no child is immunoreactive at birth; therefore, ${P}_0=0$.

### Data sources for ARI estimates

A global ARI estimate was calculated from TST surveys used to reestimate the global burden of latent TB infection by Houben and Dodd (3). This value was a simple average of ARI estimates from 141 TST surveys collected from Cauthen et al. (8) and a systematic review of the literature (3). The resulting global average ARI of 1.5% (95% confidence interval (CI): 1.3, 1.7) was used for the primary analysis. For the primary results, *k_a_* was only dependent on ARI_0_. The annual decline (2.3%) component of *k_a_* was evaluated further in the sensitivity analyses.

### Data sources for Mtb immunoreactivity test reversion

Reversion probabilities—classified per age group—were used to illustrate the degree of ARI underestimation obtained from the model. These were collected from 2 population-wide TST surveys and 1 adolescent IGRA survey. The first TST survey, by Grzybowski and Allen (11), was conducted in 1959 among 29,000 individuals of all ages in Victoria County, Ontario, Canada; it consisted of 5 consecutive annual TST surveys, in which an area of induration greater than or equal to 5 mm was considered a positive result. At the time, Bacillus Calmette-Guérin vaccination was not considered in newborns or infants and was only recommended for contacts of patients with active TB. The study provided numerators (number of reversions) and denominators (positive reactors retested in 1 year) used for age-group–specific reversion probabilities; we calculated 95% CIs for the given proportions to account for uncertainty in the probabilities. In the second TST survey, Fine et al. (10) described a set of over 64,000 TSTs performed in 2 total population surveys in the Karonga District, northern Malawi, from 1980 to 1989; TST reversion data were available from paired results from 6,991 individuals who participated in both surveys. An area of induration greater than or equal to 10 mm was considered a positive result. Reversion probabilities in females without a Bacillus Calmette-Guérin scar were presented in a plot and were extracted using a Web-based plot digitizer (22). Confidence intervals were not available, since the absolute numerator and denominator were not provided. On the other hand, in the IGRA survey, which was conducted by Andrews et al. (12) from 2005 to 2007, students aged 12–18 years were recruited from local schools in Worcester, South Africa. The age-specific annual Mtb immunoreactivity test reversion probabilities from all studies are displayed in Table 1.

**Table 1:** Age-Specific Annual *Mycobacterium tuberculosis* Immunoreactivity Test Reversion Probabilities in 3 Different Studies

**First Author, Year (Reference No.) and Age Group, years**	**Setting (Date Range)**	**Annual Reversion Probability, %**	**95% CI**
*TST Surveys*			
Grzybowski, 1964 (11)	Victoria County, Ontario, Canada (1958–1962)		
≤19		22.2	15.2, 31.4
20–39		8.0	4.9, 12.6
40–59		4.8	3.2, 6.9
≥60		9.0	6.5, 12.3
Fine, 1999 (10)	Karonga District, Malawi (1980–1989)		
≤4		17.9	
5–9		10.2	
10–14		7.5	
15–19		6.1	
20–24		5.3	
25–29		4.8	
30–39		4.1	
≥40		3.7	
*IGRA Survey*			
Andrews, 2015 (12)	Worcester, South Africa (2005–2007)		
12–18		9.9	8.8, 11.1

Abbreviations: CI, confidence interval; TST, tuberculin skin test; IGRA, interferon-γ release assay.

To test the application of the model, we used ARI estimates from 2 population-wide TST surveys as illustrative examples to calculate the difference between the observed ARIs of the studies and the true ARIs of the model. Firstly, the study by Hoa et al. (16) was a nationwide TST survey carried out in Vietnam among children aged 6–14 years from 2006 to 2007; the study produced an ARI estimate of 1.7% (95% CI: 1.5, 1.8), calculated from a TST-positive prevalence of 16.7% in a population with a mean age of 10.8 years. Secondly, the study by Wood et al. (17) was conducted among human immunodeficiency virus–negative individuals aged 5–40 years in Cape Town, South Africa. The study derived an ARI of 3.9% (95% CI: 2.2, 5.7) from an estimated TST-positive prevalence of 18.1% among 5-year-olds, an ARI of 3.9% (95% CI: 3.3, 4.5%) from an estimated prevalence of 32.7% among 10-year-olds, and an ARI of 4.8% (95% CI: 4.1, 5.5) from an estimated prevalence of 52.0% among 15-year-olds.

### Sensitivity analyses

We performed sensitivity analyses to assess the impact of the parameters on the ARI underestimation output. First, model outputs using the lower and upper bounds of the 95% CIs of the baseline ARI (1.3–1.7) were explored. Additionally, considering the heterogeneity in the global TB burden, we also used an initial ARI of 5%, accounting for high-burden settings. Moreover, the component of annual risk decrease was incorporated into parameter *k_a_*. The global annual rate of decline for TB incidence was estimated to be 2.3%, with some regions presenting more notable decreases (2).

## RESULTS

**Figure 2:**
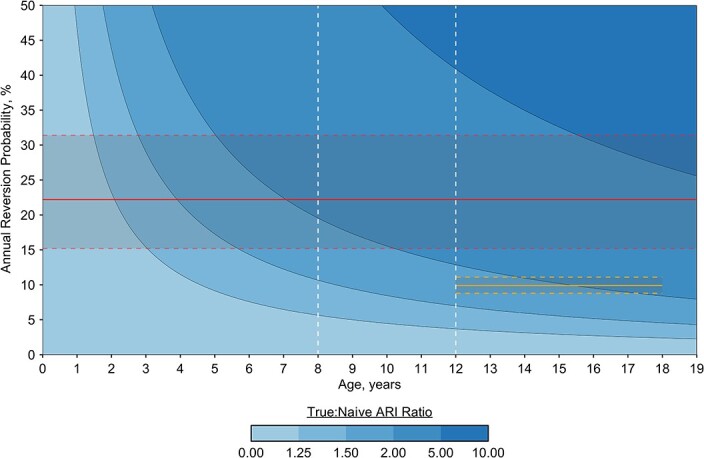
Underestimation of the annual risk of infection (ARI) with *Mycobacterium tuberculosis* according to varying annual reversion probabilities. The ratio between the true ARI (varying reversion levels) and the naive ARI (no reversion) represents the true increase in the ARI. Baseline parameters were a 1.5% ARI at birth and no decline in annual risk. Tuberculin skin test (TST) reversion probabilities (red line; dashed red lines represent 95% confidence intervals) were derived from the paper by Grzybowski and Allen (11), and interferon-γ release assay reversion probabilities (yellow line; dashed yellow lines represent 95% confidence intervals) were derived from the paper by Andrews et al. (12). White dashed lines represent the age range of populations in which most TST surveys are conducted. The study by Grzybowski and Allen (11) was conducted in Ontario, Canada (1958–1962), and the study by Andrew et al. (12) was conducted in Worcester, South Africa (2005–2007).

Figure 2 shows the degree of ARI underestimation due to ignoring reversion. In the age range 8–12 years, where most TST surveys are conducted, we found that for the TST (and in the range of reversion probabilities from Grzybowski and Allen (11) and Andrews et al. (12)), the true ARI was 2–5 times higher than that estimated under the naive scenario (i.e., assuming no reversion). With the following age-group reversion probabilities, the ARI underestimation was maintained in the older populations, rising to at least a 5-fold increase of the true ARI after age 60 years (see Web Figure 1, available at https://doi.org/10.1093/aje/kwad028). The lower observed TST reversion probabilities from Fine et al. (10) gave a 1.25- to 1.50-fold increase of the true ARI from age 3 years onwards and a more than 2-fold increase from age 12 years onwards (Web Figure 2). In the case of IGRA, the narrow reversion probabilities led to a 1.50- to 2-fold increase of the true ARI for ages 12–18 years, within the reversion probabilities from Andrews et al. (12).

Outside of the empirical reversion probabilities, Figure 2 shows how ARI underestimation grew with increasing levels of annual reversion probabilities, as well as with increasing age at which immunoreactivity was tested. Annual reversion probabilities up to 2.5% increased the true ARI by less than 1.25 times. After the first life year, changes in reversion probabilities for a particular age could reach diverse levels of underestimation (Web Figure 1).

The impact of reversion on the observed ARIs was evaluated in 2 population-wide surveys. For the study by Hoa et al. (16), the observed ARI of 1.7% at the mean age of 11 years, adjusting for reversion (using empirical reversion probabilities from Grzybowski and Allen (11)), showed the true ARI to be twice that originally observed. Likewise, in the survey by Wood et al. (17), the observed ARI of 3.9% would be increased by a factor of 1.5 at age 5 years and by a factor of 2 or more at ages 10 and 15 years (considering the empirical reversion probabilities from Grzybowski and Allen (11)).

### Sensitivity analyses

There was no notable difference between the contour maps produced by the lower and upper bounds of the 95% CI of the 1.5% baseline ARI, within the reversion probability ranges from the TST and IGRA surveys (Web Figures 3 and 4). When using a 5% baseline ARI, more discernible true ARI increases were evident at higher reversion probabilities (Web Figure 5). Incorporating the global decline in TB incidence (2.3%) into the model increased the true ARI underestimation, albeit slightly (Web Figure 6).

## DISCUSSION

We estimated that the true ARI for Mtb immunoreactivity surveys conducted in school-age children and using empirical data on TST reversion was 2–5 times higher than the baseline value that did not account for reversion. Failing to account for Mtb immunoreactivity test reversion in estimating the ARI significantly underestimates the true value, and the cumulative effect of reversion can be seen in time. In recent work, Dowdy and Behr (23) explored ARI underestimation due to increasing infection risks in adolescence and early adulthood, resistance to infection, and immunoreactivity test reversion, concluding that the latter could underestimate the risk of infection by one-third or more. In our study, we used empirical data for reversion and explored the impact across age groups in detail, highlighting how reversion is important on its own but probably differs by age and immunoreactivity test. More recent data on reversion, especially of new tools (24), are urgently needed; this is an important concept to explore and consider when interpreting future ARI estimates of recent surveys.

In the original work, Sutherland concluded that reversion probabilities above 1% would significantly impact ARI estimates (13). However, as we have seen, empirical data for TST/IGRA reversions in populations have shown that the probabilities strongly exceed 1% per year and vary by age (10–12), although the reversion probabilities are still poorly quantified and understood for new tests. Reversions may result from a myriad of different factors, including self-clearance of Mtb infection, cross-reactivity with Bacillus Calmette-Guérin vaccination or nontuberculous mycobacteria (in the case of the TST), and false-negative reactions due to impaired immune response. Note that the difference in ARI underestimation depending on the tool used might not be related to the actual tool but probably depends on the TB incidence in those settings at the time of the surveys, since the likelihood of reversion might be influenced by reinfection. Thus, lower reversion probabilities could be seen in settings with a higher risk of reinfection (25, 26). While data on reversion from novel diagnostic methods are nonexistent at present, our work highlights why it is crucial to acquire such data and how they may affect ARI estimates. Nonetheless, regardless of how—and to what extent—reversions occur, our findings focus more on the implications of the underestimation and interpretation of the resulting ARI.

Another essential issue with regard to interpretation of the ARI is its reliance on the host immune response to Mtb, which is an indirect ascertainment of Mtb infection. Because of the limitations of Mtb immunoreactivity tests, the interpretation of a positive test result as a marker of true infection—that is, harboring viable Mtb bacilli and being at risk of TB disease—is unclear. While our findings call for conscientious interpretations of the ARI given the reversion phenomenon, TB prevention and care may benefit from an improved biomarker for detecting Mtb infection that will enable more direct estimation of the true ARI. Luckily, some biomarkers are already being explored (27, 28), some providing the additional benefit of identifying individuals at higher risk of progression to active TB disease (28).

ARI estimates are key to understanding time trends in TB burden and dynamics and are important to inform subsequent policy. Given the substantial impact of reversion on ARI estimates, this naturally occurring phenomenon should be recognized in ARI calculations or, at minimum, its interpretations (29). Our exploratory analysis of the TST prevalence surveys by Hoa et al. (16) and Wood et al. (17) illustrates how the true ARI can be at least 2 times higher than the naive ARI. We may apply our understanding of the impact of ARI estimation to other existing surveys, such as India*’*s recent nationally representative survey, as reversion would mean a true transmission risk 2–5 times as high (30). Caution in interpretation of the majority of published ARIs to date is essential, including global estimates of individuals recently or remotely infected with Mtb (3).

### Limitations

The reversion probabilities used to highlight the degree of underestimation may differ by TB incidence in the setting, the immunoreactivity test, and the cutoff used. For the latter, issues arise from the use of reversion probabilities from the report of Grzybowski and Allen (11) because of the instability of the test, mainly the variability around the 5-mm single cutoff point. This issue is exacerbated by interreader variability and digit bias often encountered when using TSTs (31). In turn, the reversion probabilities from Fine et al. (10) are more convincing, as they adhere to the American Thoracic Society/Centers for Disease Control and Prevention definitions, which address this variability. Despite this, we opted to base our TST results on the reversion probabilities of Grzybowski and Allen (11), since they provide a range of uncertainty in their estimates (32). Similarly, IGRA reversions are also overemphasized in the so-called uncertainty zone (0.2–0.7 IU/ml) around the default cutoff value, where they are as high as 52%, declining as the value increases (12, 32). By presenting a wide range of reversion probabilities (up to 50%), we provide a contour map that serves as a guide that could be used to explore ARI underestimation as seen by other empirical probabilities. While our simple model with 2 binary outcomes enabled a clear analysis of the impact of reversion, it excluded other phenomena which could also play a role in Mtb immunoreactivity and, subsequently, the ARI.

The model assumed that the risks of Mtb immunoreactivity were the same for primary infections and reinfections, and while it is not possible to determine whether there would also be a reduced risk of immunoreactivity conversion (33), studies have shown a risk reduction in the progression of TB disease in previously “infected” individuals—that is, persons with positive Mtb immunoreactivity (34). Hypothetically, if we assumed that a risk reduction would be observed among individuals who had converted before, then, for the estimates accounting for reversion, the Mtb immunoreactivity prevalences—and their corresponding ARIs—would have been lower than those obtained in the primary analysis, thus resulting in a higher ratio and a greater degree of underestimation. Another phenomenon that could affect the estimated ARI is resistance to Mtb infection in some individuals (i.e., repeatedly negative Mtb immunoreactivity tests in persons who have had close contact with pulmonary TB patients, such as household contacts, miners, etc.) (35–37). Including an Mtb resistance parameter would affect the naive and true ARIs in similar ways; therefore, it would not be expected to alter the true ARI:naive ARI ratios observed in our primary results. Finally, Bacillus Calmette-Guérin vaccination and nontuberculous mycobacteria exposure are known to cause false-positive TST results (4, 5), which may contribute to a degree of overestimation when using reactivity to assess ARI. However, their contribution to reactivity and whether and how they may modify infection risks and reversion probability is unknown, so we did not include them in the model.

### Conclusions

Not accounting for reversion leads to a stark underestimation of the true ARI in populations, which changes our understanding and interpretation of Mtb transmission intensity. Considering our findings, interpretations of ARI estimates should be handled prudently. Categorization by ARI levels and mathematical models of TB disease relying on ARI as a parameter would need to be amended. Reversion probabilities specific to a region, test, and even age group are needed to increase the interpretation of ARIs from future cross-sectional surveys. Adjustment for the reversion probability and its cumulative effect with increasing age will provide a more accurate reflection of the burden and dynamics of Mtb infection.

## Supplementary Material

Web_Material_kwad028
