# Supplementary material for: Impact of Reversion of Mycobacterium tuberculosis Immunoreactivity Tests on the Estimated Annual Risk of Tuberculosis Infection
Source: Am J Epidemiol. 2023 Feb 7;192(12):1937–43. doi: 10.1093/aje/kwad028 (PMC10691197; doi:10.1093/aje/kwad028)
Supplement: Web_Material_kwad028 [file web_material_kwad028.zip › Web_Material_kwad028.pdf]

WEB MATERIAL

Impact of Reversion of *Mycobacterium tuberculosis* Immunoreactivity Tests on the Estimated Annual Risk of Tuberculosis Infection

Alvaro Schwalb[1,2,3], Jon C. Emery[1,2], Katie D. Dale[4], Katherine C. Horton[1,2], César A. Ugarte-Gil[3,5], and Rein M. G. J. Houben[1,2]

[1] TB Modeling Group, TB Centre, London School of Hygiene and Tropical Medicine, London, United Kingdom.

[2] Department of Infectious Disease Epidemiology, London School of Hygiene and Tropical Medicine, London, United Kingdom.

[3] Instituto de Medicina Tropical Alexander von Humboldt, Universidad Peruana Cayetano Heredia, Lima, Peru.

[4] Victorian Tuberculosis Program, Melbourne Health, Melbourne, Victoria, Australia.

[5] TB Centre, London School of Hygiene and Tropical Medicine, London, United Kingdom.

Table of Contents

Web Figure 1 …………………………………………………………………………………... 2
Web Figure 2 ……………………………………………………………………………….… 3
Web Figure 3 ……………………………………………………………………………….… 4
Web Figure 4 ………………………………………………………………………………….. 5
Web Figure 5 ……………………………………………………………………………….… 6
Web Figure 6 ………………………………………………………………………………….. 7
Web References …………………………………………………………………………….. 8

Web Figure 1

Contour maps of ARI underestimation by varying annual reversion probabilities.

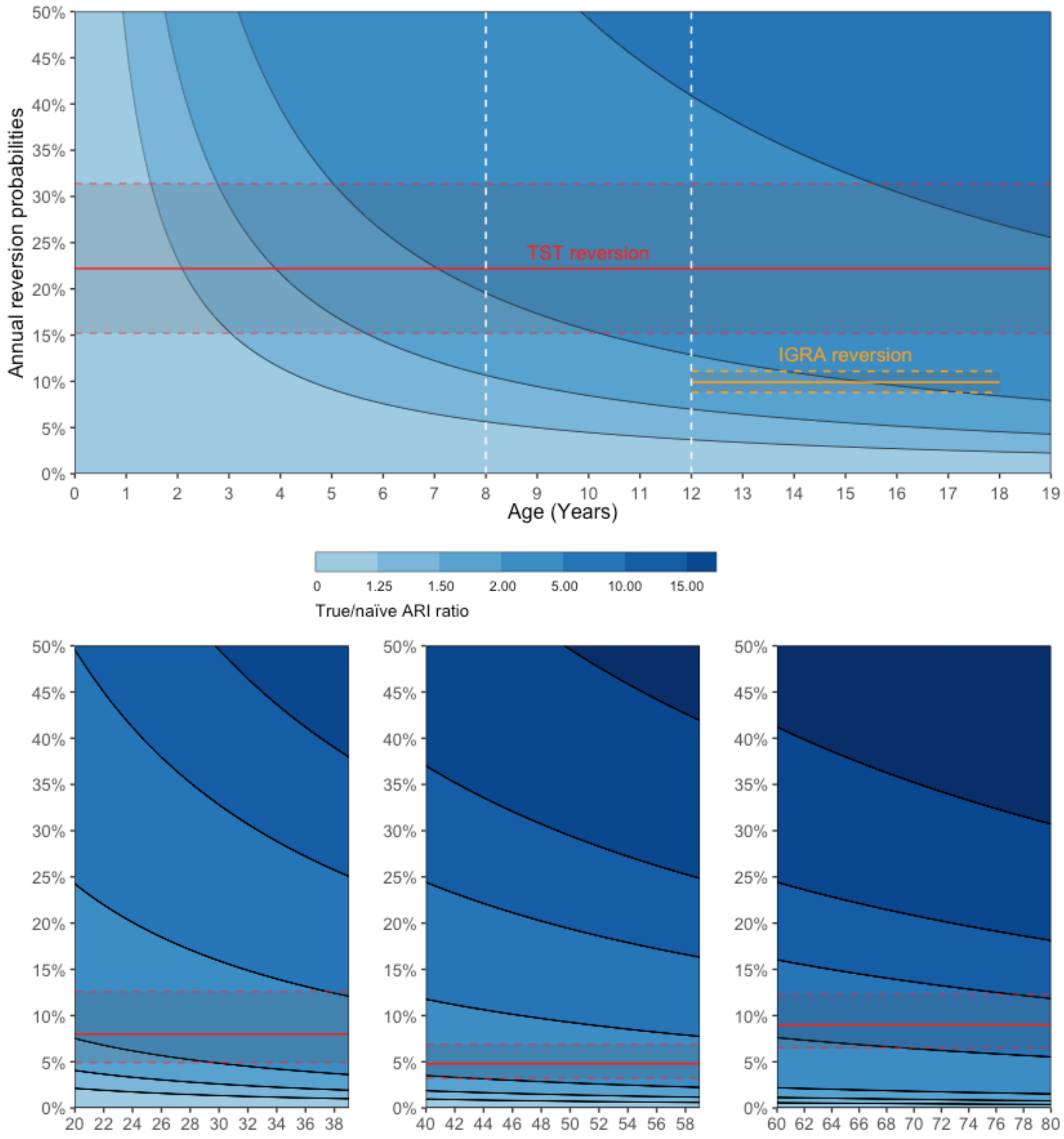

The ratio between true (varying reversion levels) and naïve ARI (no reversion) represents true ARI increase. Baseline parameters: 1.5% ARI at birth and no decline in annual risk. TST reversion probabilities from Grzybowski and Allen (represented by the red line; dotted red lines represent 95%CI) and IGRA reversion probabilities from Andrews et al. (represented by the yellow line; dotted yellow lines represent 95%CI)(1,2). White dotted lines represent the age range of populations where most TST surveys are conducted. TST, Tuberculin skin test; IGRA, Interferon-gamma release assay.

Web Figure 2

Contour map of ARI underestimation by varying annual reversion probabilities.

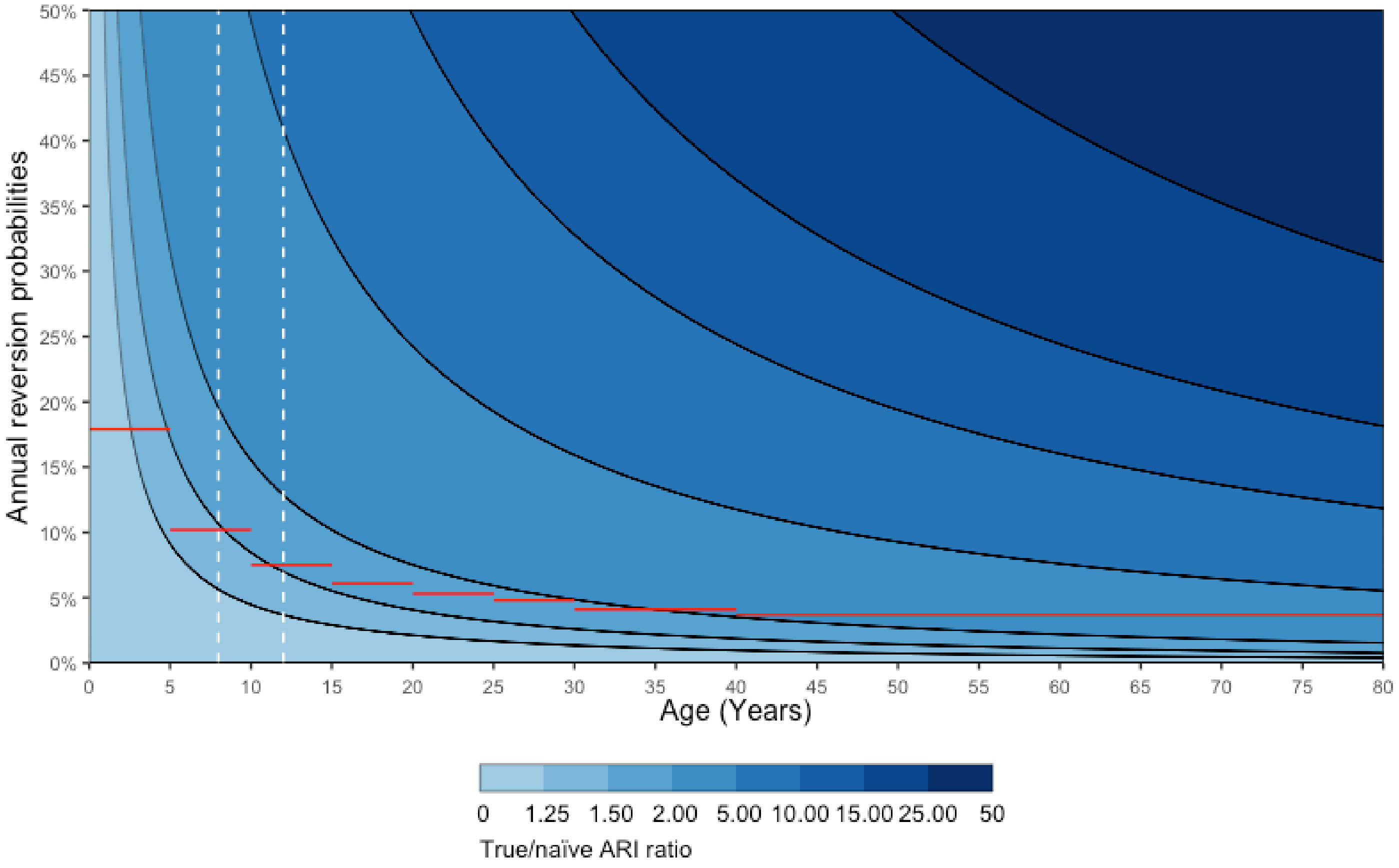

The ratio between true (varying reversion levels) and naïve ARI (no reversion) represents true ARI increase. Baseline parameters: 1.5% ARI at birth and no decline in annual risk. Age-specific TST reversion probabilities from Fine et al. (represented by the red lines)(3). White dotted lines represent age range of populations where most TST surveys are conducted. TST, Tuberculin skin test.

Web Figure 3

Contour map of ARI underestimation by varying annual reversion probabilities.

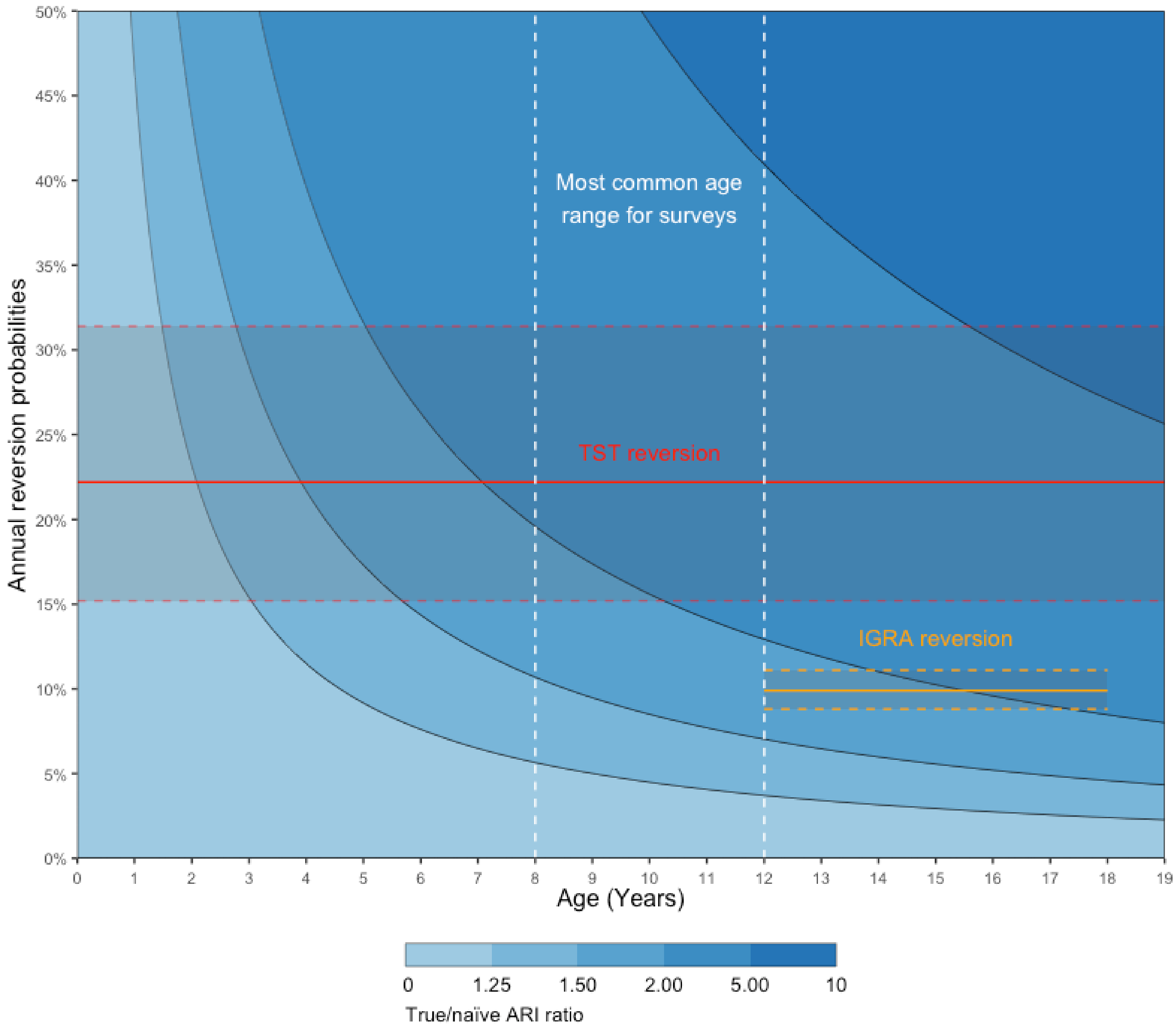

The ratio between true (varying reversion levels) and naïve ARI (no reversion) represents true ARI increase. Baseline parameters: ***1.3% ARI at birth and no decline in annual risk.*** *TST reversion probabilities from Grzybowski and Allen (represented by the red line; dotted red lines represent 95%CI) and IGRA reversion probabilities from Andrews et al. (represented by the yellow line; dotted yellow lines represent 95%CI)(1,2). White dotted lines represent age range of populations where most TST surveys are conducted. TST, Tuberculin skin test; IGRA, Interferon-gamma release assay.*

Web Figure 4

Contour map of ARI underestimation by varying annual reversion probabilities.

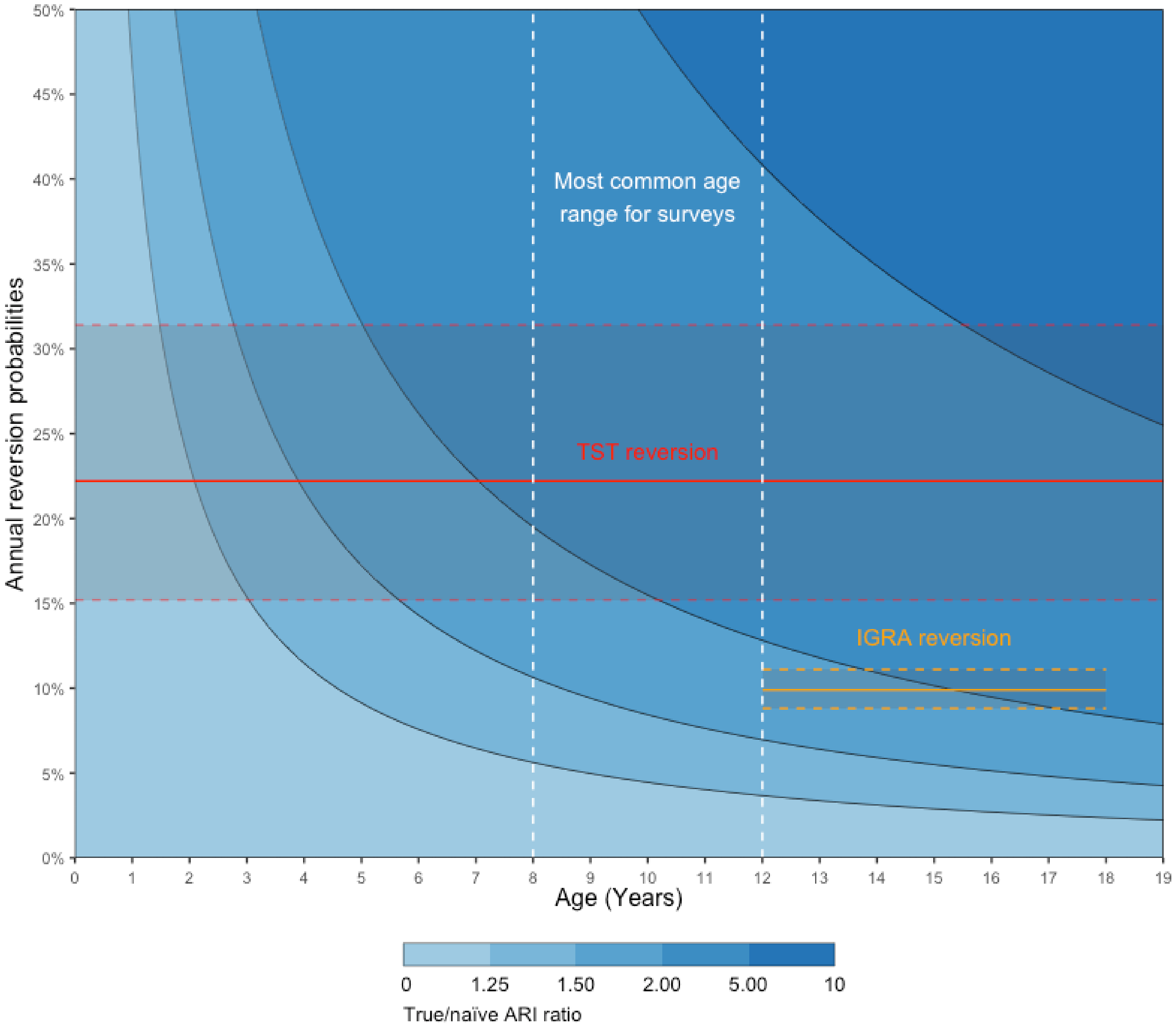

The ratio between true (varying reversion levels) and naïve ARI (no reversion) represents true ARI increase. Baseline parameters: ***1.7% ARI at birth and no decline in annual risk.*** *TST reversion probabilities from Grzybowski and Allen (represented by the red line; dotted red lines represent 95%CI) and IGRA reversion probabilities from Andrews et al. (represented by the yellow line; dotted yellow lines represent 95%CI)(1,2). White dotted lines represent age range of populations where most TST surveys are conducted. TST, Tuberculin skin test; IGRA, Interferon-gamma release assay.*

Web Figure 5

Contour map of ARI underestimation by varying annual reversion probabilities.

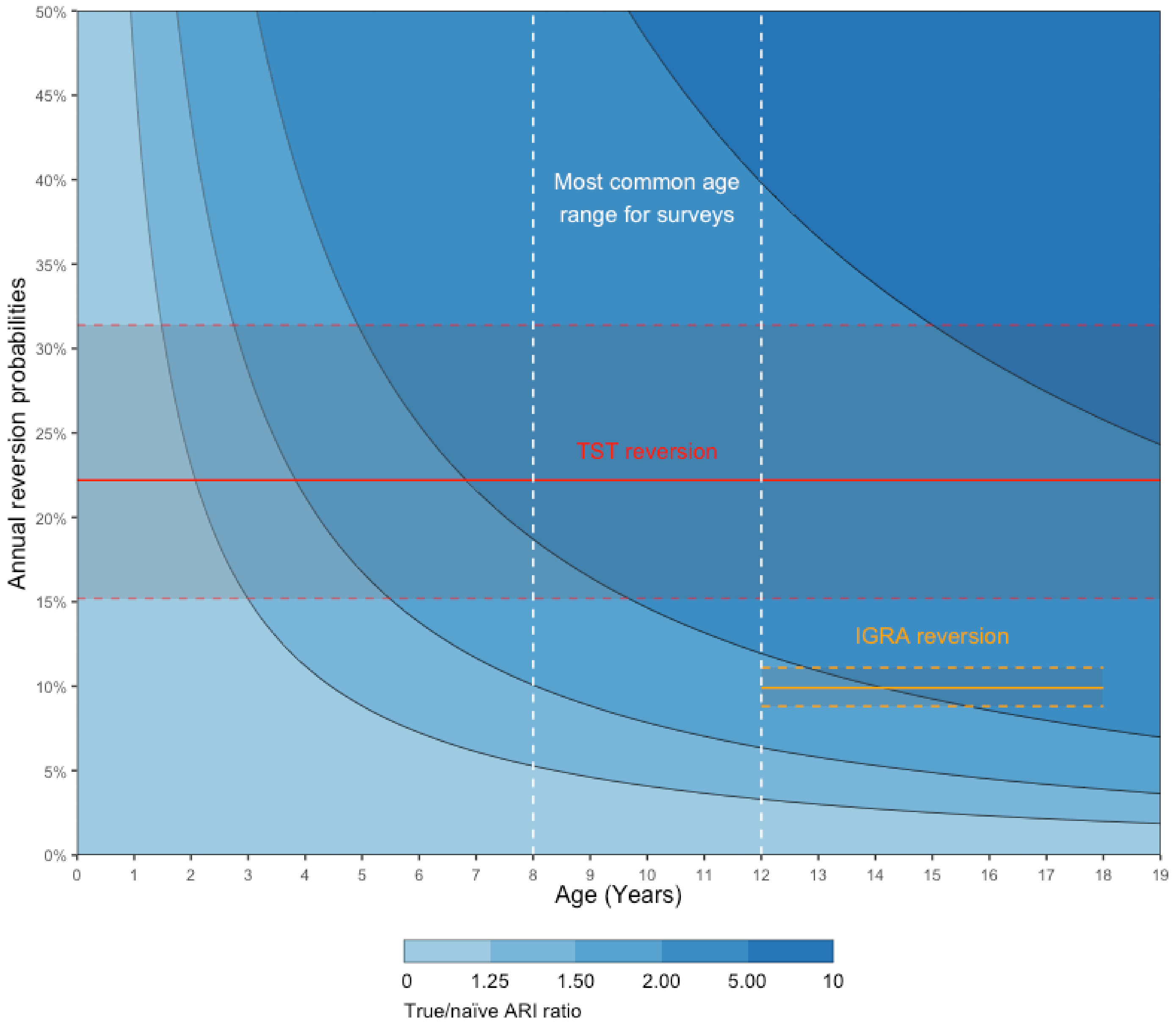

The ratio between true (varying reversion levels) and naïve ARI (no reversion) represents true ARI increase. Baseline parameters: ***5.0% ARI at birth and no decline in annual risk.*** *TST reversion probabilities from Grzybowski and Allen (represented by the red line; dotted red lines represent 95%CI) and IGRA reversion probabilities from Andrews et al. (represented by the yellow line; dotted yellow lines represent 95%CI)(1,2). White dotted lines represent age range of populations where most TST surveys are conducted. TST, Tuberculin skin test; IGRA, Interferon-gamma release assay.*

Web Figure 6

Contour map of ARI underestimation by varying annual reversion probabilities.

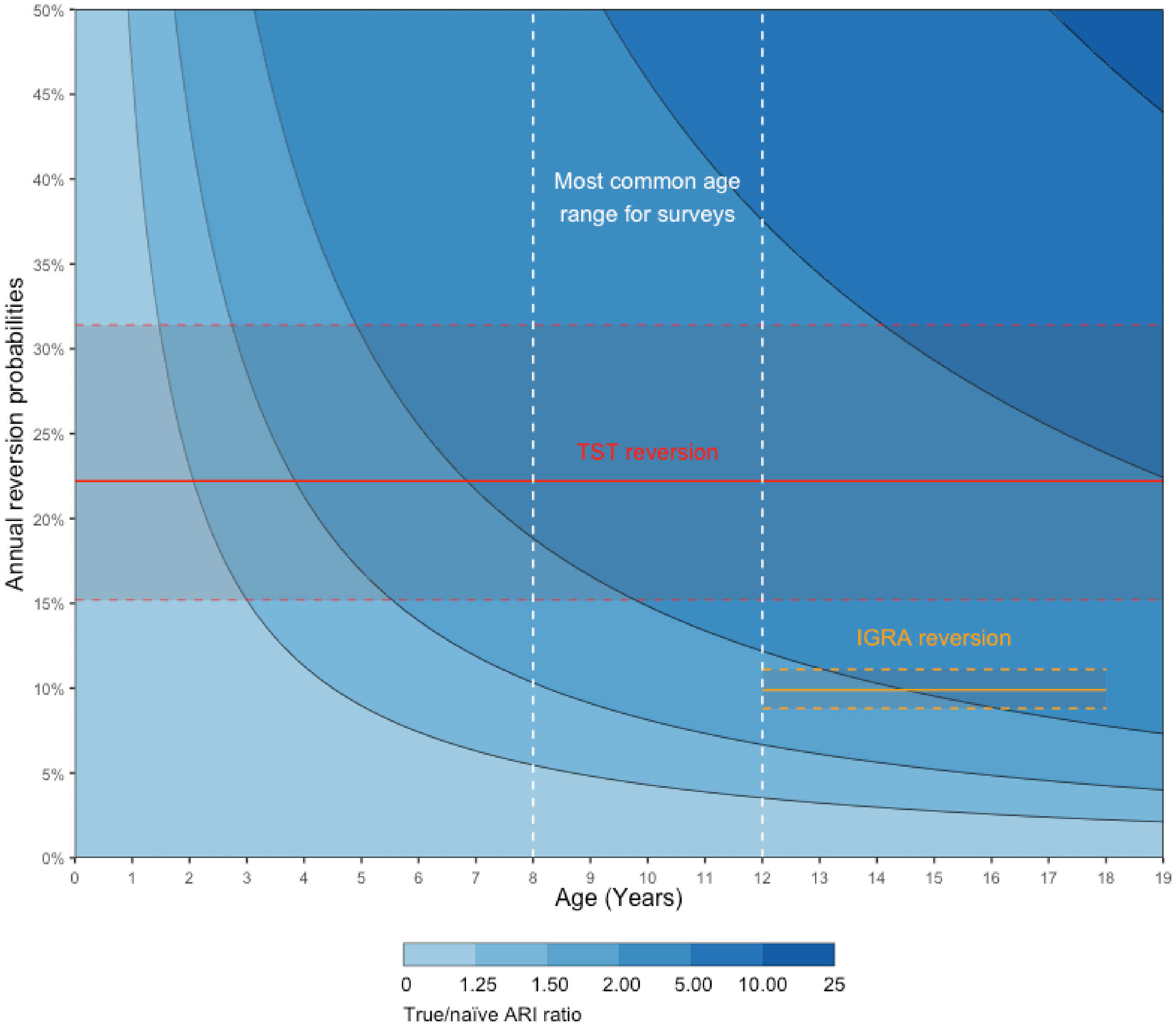

The ratio between true (varying reversion levels) and naïve ARI (no reversion) represents true ARI increase. Baseline parameters: ***1.5% ARI at birth and 2.3% decline in annual risk.*** *TST reversion probabilities from Grzybowski and Allen (represented by the red line; dotted red lines represent 95%CI) and IGRA reversion probabilities from Andrews et al. (represented by the yellow line; dotted yellow lines represent 95%CI)(1,2). White dotted lines represent age range of populations where most TST surveys are conducted. TST, Tuberculin skin test; IGRA, Interferon-gamma release assay.*

Web References

1. Grzybowski S, Allen EA. The challenge of tuberculosis in decline: A study based on the epidemiology of tuberculosis in Ontario, Canada. *Am. Rev. Respir. Dis.* 1964;90:707–720.
2. Andrews JR, Hatherill M, Mahomed H, et al. The dynamics of QuantiFERON-TB gold in-tube conversion and reversion in a cohort of South African adolescents. *Am. J. Respir. Crit. Care Med.* 2015;191(5):584–591.
3. Fine PE, Bruce J, Ponnighaus JM, et al. Tuberculin sensitivity: conversions and reversions in a rural African population. *Int. J. Tuberc. Lung Dis.* 1999;3(11):962–975.
